# {4-Bromo-2-[(2-sulfido­phen­yl)imino­meth­yl]phenolato-κ^3^ *S*,*N*,*O*}(triphenyl­phosphane-κ*P*)nickel(II)

**DOI:** 10.1107/S1600536811008907

**Published:** 2011-03-12

**Authors:** M. Muthu Tamizh, R. Karvembu, B. Varghese, Edward R. T. Tiekink

**Affiliations:** a Department of Chemistry, National Institute of Technology, Tiruchirappalli 620 015, India; b Sophisticated Analytical Instruments Facility, Indian Institute of Technology-Madras, Chennai 600 036, India; c Department of Chemistry, University of Malaya, 50603 Kuala Lumpur, Malaysia

## Abstract

The Ni^II^ atom in the title complex, [Ni(C_13_H_8_BrNOS)(C_18_H_15_P)], is coordinated by the N, O and S atoms of the dianionic tridentate ligand, and its square-planar geometry is completed by a phosphane P atom. The dihedral angle between the aromatic rings in the 4-bromo-2-[(2-sulfido­phen­yl)imino­meth­yl]phenolate ligand is 2.01 (14)°. The most prominent feature of the packing is the presence of supra­molecular chains aligned along the *a* axis, mediated by C—H⋯S inter­actions.

## Related literature

For chemical background and related structures, see: Muthu Tamizh *et al.* (2009 ▶).
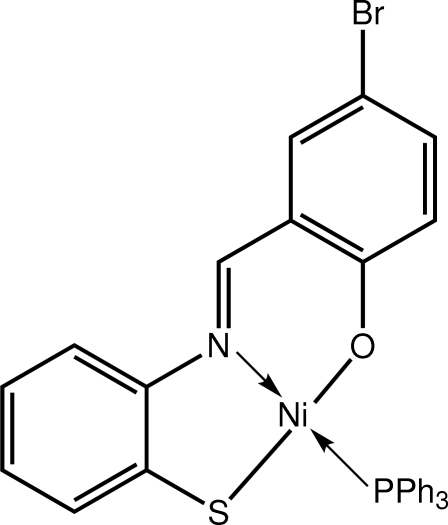


## Experimental

### 

#### Crystal data

[Ni(C_13_H_8_BrNOS)(C_18_H_15_P)]
*M* *_r_* = 627.15
Orthorhombic, 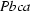

*a* = 9.7197 (2) Å
*b* = 18.7729 (6) Å
*c* = 29.8372 (7) Å
*V* = 5444.3 (2) Å^3^
*Z* = 8
Mo *K*α radiation
μ = 2.34 mm^−1^
*T* = 293 K
0.25 × 0.20 × 0.15 mm

#### Data collection

Bruker Kappa APEXII CCD diffractometer
Absorption correction: multi-scan (*SADABS*; Sheldrick, 1996 ▶) *T*_min_ = 0.592, *T*_max_ = 0.720
31370 measured reflections
6260 independent reflections
4184 reflections with *I* > 2σ(*I*)
*R*_int_ = 0.050

#### Refinement

*R*[*F* ^2^ > 2σ(*F* ^2^)] = 0.035
*wR*(*F* ^2^) = 0.092
*S* = 1.03
6260 reflections
334 parameters
H-atom parameters constrained
Δρ_max_ = 0.42 e Å^−3^
Δρ_min_ = −0.43 e Å^−3^

### 

Data collection: *APEX2* (Bruker, 2004 ▶); cell refinement: *SAINT* (Bruker, 2004 ▶); data reduction: *SAINT*; program(s) used to solve structure: *SHELXS97* (Sheldrick, 2008 ▶); program(s) used to refine structure: *SHELXL97* (Sheldrick, 2008 ▶); molecular graphics: *ORTEP-3* (Farrugia, 1997 ▶) and *DIAMOND* (Brandenburg, 2006 ▶); software used to prepare material for publication: *publCIF* (Westrip, 2010 ▶).

## Supplementary Material

Crystal structure: contains datablocks global, I. DOI: 10.1107/S1600536811008907/hb5813sup1.cif

Structure factors: contains datablocks I. DOI: 10.1107/S1600536811008907/hb5813Isup2.hkl

Additional supplementary materials:  crystallographic information; 3D view; checkCIF report

## Figures and Tables

**Table 1:** Selected bond lengths (Å)

Ni—S1	2.1219 (7)
Ni—P1	2.1975 (7)
Ni—O1	1.8494 (18)
Ni—N1	1.900 (2)

**Table 2:** Hydrogen-bond geometry (Å, °)

*D*—H⋯*A*	*D*—H	H⋯*A*	*D*⋯*A*	*D*—H⋯*A*
C23—H23⋯S1^i^	0.93	2.84	3.620 (3)	142

Symmetry code: (i) 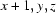.

**Fig. 1.**
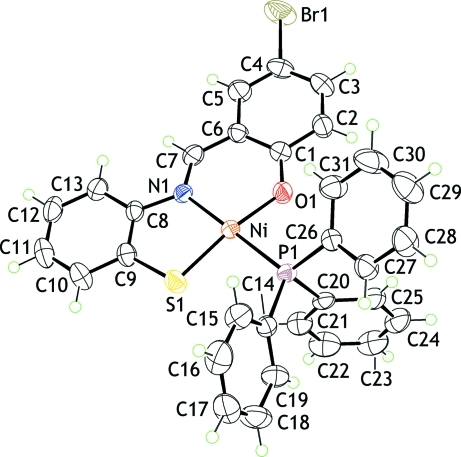
The molecular structure of (I) showing displacement ellipsoids at the 35% probability level.

**Fig. 2.**
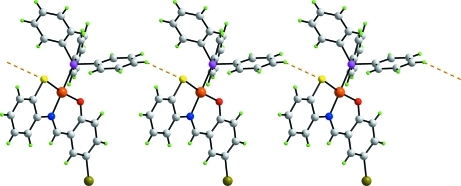
Supramolecular chain aligned along the a axis in (I) mediated by C—H···S contacts shown as orange dashed lines.

## supplementary crystallographic information

### Comment

The title complex, (I), was investigated as part of a wider investigation of complexes of interest owing to their relationship to Ni/Fe hydrogenases (Muthu Tamizh *et al.*, 2009). The Ni^II^ atom exists within a square planar donor set defined by the *N*,*O*,*S* atoms of the dinegative tridentate ligand, and a P atom derived from the phosphane ligand. The S1, P1, O1 and N1 atoms deviate -0.0535 (7), 0.0556 (8), -0.0609 (8) and 0.0588 (8) Å, from their least-squares plane, respectively (r.m.s. deviation = 0.0573 Å), and the Ni atom lies 0.0548 (9) Å out of the plane. The geometric parameters about the Ni^II^ atom, Table 1, match closely those in the parent complex (Muthu Tamizh *et al.*, 2009).

The most prominent interactions in the crystal structure are of the type C—H···S, Table 2. These connect molecules into linear supramolecular chains along the *a* axis, Fig. 2.

### Experimental

Complex (I) was prepared from the reaction between [NiCl_2_(PPh_3_)_2_] and *N*-(2-mercaptophenyl)-4-bromosalicylideneimine in ethanol solution following the literature procedure (Muthu Tamizh *et al.*, 2009). Brown prisms of (I) were obtained by the diffusion of diethyl ether vapour into its dichloromethane solution. Characterization data are as given in Muthu Tamizh *et al.* (2009).

### Refinement

The H-atoms were placed in calculated positions (C—H 0.93 Å) and were included in the refinement in the riding model approximation, with *U*_iso_(H) set to 1.2*U*_equiv_(C). A reflection, *i.e*. (2 0 0), was omitted from the final refinement owing to poor agreement.

### Crystal data

**Table:**

[Ni(C_13_H_8_BrNOS)(C_18_H_15_P)]	*F*(000) = 2544
*M**_r_* = 627.15	*D*_x_ = 1.530 Mg m^−^^3^
Orthorhombic, *P**b**c**a*	Mo *K*α radiation, λ = 0.71073 Å
Hall symbol: -P 2ac 2ab	Cell parameters from 5504 reflections
*a* = 9.7197 (2) Å	θ = 2.2–25.0°
*b* = 18.7729 (6) Å	µ = 2.34 mm^−^^1^
*c* = 29.8372 (7) Å	*T* = 293 K
*V* = 5444.3 (2) Å^3^	Prism, brown
*Z* = 8	0.25 × 0.20 × 0.15 mm

### Data collection

**Table:**

Bruker Kappa APEXII CCD diffractometer	6260 independent reflections
Radiation source: fine-focus sealed tube	4184 reflections with *I* > 2σ(*I*)
graphite	*R*_int_ = 0.050
ω and φ scans	θ_max_ = 27.5°, θ_min_ = 2.3°
Absorption correction: multi-scan (*SADABS*; Sheldrick, 1996)	*h* = −12→12
*T*_min_ = 0.592, *T*_max_ = 0.720	*k* = −13→24
31370 measured reflections	*l* = −31→38

### Refinement

**Table:**

Refinement on *F*^2^	Primary atom site location: structure-invariant direct methods
Least-squares matrix: full	Secondary atom site location: difference Fourier map
*R*[*F*^2^ > 2σ(*F*^2^)] = 0.035	Hydrogen site location: inferred from neighbouring sites
*wR*(*F*^2^) = 0.092	H-atom parameters constrained
*S* = 1.03	*w* = 1/[σ^2^(*F*_o_^2^) + (0.0435*P*)^2^ + 0.9841*P*] where *P* = (*F*_o_^2^ + 2*F*_c_^2^)/3
6260 reflections	(Δ/σ)_max_ = 0.002
334 parameters	Δρ_max_ = 0.42 e Å^−^^3^
0 restraints	Δρ_min_ = −0.43 e Å^−^^3^

### Special details

**Table:**

Geometry. All s.u.'s (except the s.u. in the dihedral angle between two l.s. planes) are estimated using the full covariance matrix. The cell s.u.'s are taken into account individually in the estimation of s.u.'s in distances, angles and torsion angles; correlations between s.u.'s in cell parameters are only used when they are defined by crystal symmetry. An approximate (isotropic) treatment of cell s.u.'s is used for estimating s.u.'s involving l.s. planes.
Refinement. Refinement of *F*^2^ against ALL reflections. The weighted *R*-factor *wR* and goodness of fit *S* are based on *F*^2^, conventional *R*-factors *R* are based on *F*, with *F* set to zero for negative *F*^2^. The threshold expression of *F*^2^ > 2σ(*F*^2^) is used only for calculating *R*-factors(gt) *etc*. and is not relevant to the choice of reflections for refinement. *R*-factors based on *F*^2^ are statistically about twice as large as those based on *F*, and *R*- factors based on ALL data will be even larger.

### Fractional atomic coordinates and isotropic or equivalent isotropic displacement parameters (Å^2^)

**Table:**

	*x*	*y*	*z*	*U*_iso_*/*U*_eq_	
Ni	0.70528 (3)	0.093546 (17)	0.666881 (10)	0.02872 (9)	
Br1	0.89896 (5)	−0.08458 (2)	0.874812 (12)	0.07450 (14)	
P1	0.79107 (6)	0.06542 (4)	0.60108 (2)	0.02893 (15)	
S1	0.57705 (7)	0.17098 (4)	0.63624 (2)	0.04198 (18)	
O1	0.83786 (18)	0.03231 (10)	0.68907 (6)	0.0403 (5)	
N1	0.6293 (2)	0.11848 (11)	0.72343 (7)	0.0298 (5)	
C1	0.8475 (3)	0.00804 (14)	0.73001 (8)	0.0337 (6)	
C2	0.7685 (3)	0.03472 (14)	0.76579 (8)	0.0344 (6)	
C3	0.7875 (3)	0.00663 (16)	0.80893 (9)	0.0463 (7)	
H3	0.7363	0.0244	0.8327	0.056*	
C4	0.8795 (3)	−0.04598 (16)	0.81620 (9)	0.0465 (7)	
C5	0.9575 (3)	−0.07331 (16)	0.78142 (10)	0.0466 (7)	
H5	1.0204	−0.1096	0.7868	0.056*	
C6	0.9417 (3)	−0.04669 (16)	0.73927 (9)	0.0441 (7)	
H6	0.9945	−0.0652	0.7161	0.053*	
C7	0.6686 (3)	0.08912 (15)	0.76043 (9)	0.0393 (7)	
H7	0.6266	0.1055	0.7865	0.047*	
C8	0.5279 (2)	0.17371 (13)	0.72504 (8)	0.0309 (6)	
C9	0.4973 (3)	0.20480 (14)	0.68419 (9)	0.0357 (6)	
C10	0.4033 (3)	0.26043 (16)	0.68227 (11)	0.0488 (8)	
H10	0.3848	0.2825	0.6550	0.059*	
C11	0.3372 (3)	0.28300 (17)	0.72053 (12)	0.0523 (8)	
H11	0.2738	0.3200	0.7191	0.063*	
C12	0.3653 (3)	0.25079 (16)	0.76073 (11)	0.0491 (8)	
H12	0.3195	0.2656	0.7865	0.059*	
C13	0.4604 (3)	0.19692 (15)	0.76332 (10)	0.0435 (7)	
H13	0.4796	0.1759	0.7908	0.052*	
C14	0.7096 (3)	0.10195 (14)	0.55127 (8)	0.0320 (6)	
C15	0.5778 (3)	0.07925 (18)	0.54121 (10)	0.0488 (8)	
H15	0.5358	0.0448	0.5589	0.059*	
C16	0.5086 (4)	0.1074 (2)	0.50515 (11)	0.0619 (10)	
H16	0.4202	0.0915	0.4986	0.074*	
C17	0.5675 (4)	0.1582 (2)	0.47883 (10)	0.0582 (9)	
H17	0.5187	0.1778	0.4550	0.070*	
C18	0.6985 (4)	0.18015 (19)	0.48761 (10)	0.0607 (9)	
H18	0.7402	0.2139	0.4693	0.073*	
C19	0.7700 (3)	0.15220 (16)	0.52392 (9)	0.0456 (7)	
H19	0.8592	0.1675	0.5298	0.055*	
C20	0.9652 (3)	0.10013 (14)	0.59933 (8)	0.0329 (6)	
C21	0.9851 (3)	0.17007 (17)	0.61177 (9)	0.0460 (7)	
H21	0.9106	0.1974	0.6212	0.055*	
C22	1.1149 (3)	0.1999 (2)	0.61037 (11)	0.0600 (9)	
H22	1.1273	0.2474	0.6184	0.072*	
C23	1.2250 (3)	0.1599 (2)	0.59722 (11)	0.0605 (10)	
H23	1.3123	0.1801	0.5962	0.073*	
C24	1.2070 (3)	0.0902 (2)	0.58564 (11)	0.0566 (9)	
H24	1.2824	0.0630	0.5768	0.068*	
C25	1.0773 (3)	0.05948 (17)	0.58688 (9)	0.0429 (7)	
H25	1.0659	0.0118	0.5794	0.052*	
C26	0.8064 (2)	−0.02845 (14)	0.58757 (9)	0.0333 (6)	
C27	0.8427 (3)	−0.04952 (16)	0.54450 (9)	0.0444 (7)	
H27	0.8529	−0.0155	0.5221	0.053*	
C28	0.8637 (3)	−0.12050 (18)	0.53468 (11)	0.0544 (8)	
H28	0.8893	−0.1342	0.5059	0.065*	
C29	0.8465 (4)	−0.17076 (19)	0.56755 (12)	0.0638 (9)	
H29	0.8622	−0.2186	0.5612	0.077*	
C30	0.8064 (4)	−0.15059 (18)	0.60965 (12)	0.0675 (10)	
H30	0.7932	−0.1851	0.6316	0.081*	
C31	0.7852 (3)	−0.07989 (16)	0.61992 (10)	0.0500 (8)	
H31	0.7567	−0.0669	0.6485	0.060*	

### Atomic displacement parameters (Å^2^)

**Table:**

	*U*^11^	*U*^22^	*U*^33^	*U*^12^	*U*^13^	*U*^23^
Ni	0.03059 (18)	0.03009 (19)	0.02548 (16)	0.00211 (14)	0.00219 (13)	0.00157 (13)
Br1	0.1097 (3)	0.0692 (3)	0.0446 (2)	0.0162 (2)	−0.01411 (19)	0.02072 (17)
P1	0.0301 (3)	0.0312 (4)	0.0255 (3)	0.0001 (3)	0.0013 (3)	0.0021 (3)
S1	0.0511 (4)	0.0426 (4)	0.0323 (3)	0.0150 (3)	−0.0035 (3)	0.0012 (3)
O1	0.0441 (11)	0.0478 (12)	0.0289 (10)	0.0149 (9)	0.0042 (8)	0.0023 (9)
N1	0.0296 (11)	0.0289 (12)	0.0309 (11)	−0.0015 (9)	0.0043 (9)	0.0000 (9)
C1	0.0369 (15)	0.0320 (15)	0.0323 (14)	−0.0011 (12)	−0.0036 (11)	−0.0001 (11)
C2	0.0415 (15)	0.0318 (15)	0.0300 (13)	0.0036 (12)	−0.0003 (11)	0.0014 (11)
C3	0.0606 (19)	0.0489 (19)	0.0295 (14)	0.0086 (16)	0.0009 (13)	0.0012 (13)
C4	0.0621 (19)	0.0423 (18)	0.0351 (15)	−0.0011 (15)	−0.0110 (14)	0.0101 (13)
C5	0.0507 (18)	0.0383 (18)	0.0508 (18)	0.0080 (14)	−0.0128 (14)	0.0050 (14)
C6	0.0450 (17)	0.0453 (18)	0.0421 (16)	0.0120 (14)	0.0007 (13)	0.0014 (13)
C7	0.0464 (17)	0.0430 (17)	0.0285 (14)	0.0029 (13)	0.0079 (12)	−0.0016 (12)
C8	0.0274 (13)	0.0264 (14)	0.0389 (14)	0.0000 (11)	0.0035 (11)	−0.0038 (11)
C9	0.0341 (14)	0.0324 (15)	0.0407 (15)	0.0030 (12)	−0.0017 (12)	−0.0062 (12)
C10	0.0487 (18)	0.0434 (19)	0.0544 (18)	0.0142 (15)	−0.0116 (14)	−0.0030 (15)
C11	0.0397 (17)	0.0408 (19)	0.076 (2)	0.0116 (14)	−0.0040 (16)	−0.0111 (17)
C12	0.0431 (17)	0.0450 (19)	0.059 (2)	0.0051 (15)	0.0163 (14)	−0.0118 (16)
C13	0.0432 (17)	0.0424 (18)	0.0448 (16)	0.0031 (14)	0.0110 (13)	−0.0004 (13)
C14	0.0357 (14)	0.0366 (15)	0.0235 (12)	0.0051 (12)	0.0001 (10)	−0.0026 (10)
C15	0.0418 (17)	0.063 (2)	0.0418 (16)	−0.0044 (15)	−0.0043 (13)	0.0031 (15)
C16	0.0436 (18)	0.096 (3)	0.0466 (19)	0.0106 (18)	−0.0128 (15)	−0.0068 (19)
C17	0.072 (2)	0.075 (3)	0.0278 (15)	0.028 (2)	−0.0132 (15)	−0.0050 (16)
C18	0.084 (3)	0.061 (2)	0.0368 (17)	0.006 (2)	−0.0048 (16)	0.0128 (15)
C19	0.0537 (18)	0.049 (2)	0.0337 (15)	−0.0011 (15)	−0.0042 (13)	0.0062 (13)
C20	0.0323 (14)	0.0420 (17)	0.0244 (12)	−0.0034 (12)	−0.0006 (10)	0.0085 (11)
C21	0.0437 (17)	0.0475 (19)	0.0469 (17)	−0.0077 (15)	−0.0032 (13)	0.0015 (14)
C22	0.059 (2)	0.059 (2)	0.062 (2)	−0.0223 (18)	−0.0125 (17)	0.0061 (17)
C23	0.0377 (19)	0.087 (3)	0.057 (2)	−0.0201 (19)	−0.0117 (15)	0.0171 (19)
C24	0.0297 (16)	0.087 (3)	0.0533 (19)	0.0026 (17)	0.0003 (13)	0.0175 (18)
C25	0.0379 (16)	0.0512 (19)	0.0397 (16)	0.0019 (14)	0.0009 (12)	0.0086 (14)
C26	0.0319 (14)	0.0318 (15)	0.0363 (14)	0.0014 (11)	−0.0003 (11)	−0.0012 (11)
C27	0.0550 (18)	0.0418 (19)	0.0363 (15)	0.0096 (14)	−0.0010 (13)	−0.0039 (13)
C28	0.070 (2)	0.053 (2)	0.0401 (16)	0.0139 (17)	−0.0052 (15)	−0.0130 (16)
C29	0.091 (3)	0.0376 (19)	0.063 (2)	0.0076 (19)	−0.013 (2)	−0.0117 (17)
C30	0.109 (3)	0.0338 (19)	0.060 (2)	−0.0059 (19)	0.001 (2)	0.0049 (16)
C31	0.070 (2)	0.0369 (18)	0.0434 (17)	−0.0061 (16)	0.0073 (15)	−0.0029 (13)

### Geometric parameters (Å, °)

**Table:**

Ni—S1	2.1219 (7)	C14—C19	1.378 (4)
Ni—P1	2.1975 (7)	C14—C15	1.383 (4)
Ni—O1	1.8494 (18)	C15—C16	1.374 (4)
Ni—N1	1.900 (2)	C15—H15	0.9300
Br1—C4	1.902 (3)	C16—C17	1.362 (5)
P1—C26	1.814 (3)	C16—H16	0.9300
P1—C20	1.815 (3)	C17—C18	1.364 (4)
P1—C14	1.818 (2)	C17—H17	0.9300
S1—C9	1.747 (3)	C18—C19	1.390 (4)
O1—C1	1.307 (3)	C18—H18	0.9300
N1—C7	1.292 (3)	C19—H19	0.9300
N1—C8	1.431 (3)	C20—C21	1.378 (4)
C1—C6	1.404 (4)	C20—C25	1.381 (4)
C1—C2	1.407 (3)	C21—C22	1.381 (4)
C2—C3	1.403 (4)	C21—H21	0.9300
C2—C7	1.418 (4)	C22—C23	1.365 (5)
C3—C4	1.350 (4)	C22—H22	0.9300
C3—H3	0.9300	C23—C24	1.365 (5)
C4—C5	1.384 (4)	C23—H23	0.9300
C5—C6	1.362 (4)	C24—C25	1.387 (4)
C5—H5	0.9300	C24—H24	0.9300
C6—H6	0.9300	C25—H25	0.9300
C7—H7	0.9300	C26—C31	1.381 (4)
C8—C9	1.384 (4)	C26—C27	1.390 (4)
C8—C13	1.388 (3)	C27—C28	1.379 (4)
C9—C10	1.389 (4)	C27—H27	0.9300
C10—C11	1.376 (4)	C28—C29	1.371 (5)
C10—H10	0.9300	C28—H28	0.9300
C11—C12	1.371 (4)	C29—C30	1.369 (5)
C11—H11	0.9300	C29—H29	0.9300
C12—C13	1.372 (4)	C30—C31	1.377 (4)
C12—H12	0.9300	C30—H30	0.9300
C13—H13	0.9300	C31—H31	0.9300
			
O1—Ni—N1	96.09 (8)	C8—C13—H13	119.9
O1—Ni—S1	171.63 (6)	C19—C14—C15	118.5 (3)
N1—Ni—S1	89.17 (7)	C19—C14—P1	123.8 (2)
O1—Ni—P1	84.61 (6)	C15—C14—P1	117.7 (2)
N1—Ni—P1	179.29 (7)	C16—C15—C14	120.3 (3)
S1—Ni—P1	90.14 (3)	C16—C15—H15	119.8
C26—P1—C20	105.42 (12)	C14—C15—H15	119.8
C26—P1—C14	102.74 (12)	C17—C16—C15	121.0 (3)
C20—P1—C14	104.33 (12)	C17—C16—H16	119.5
C26—P1—Ni	117.59 (9)	C15—C16—H16	119.5
C20—P1—Ni	107.06 (9)	C16—C17—C18	119.6 (3)
C14—P1—Ni	118.32 (8)	C16—C17—H17	120.2
C9—S1—Ni	99.01 (9)	C18—C17—H17	120.2
C1—O1—Ni	126.94 (16)	C17—C18—C19	120.1 (3)
C7—N1—C8	118.9 (2)	C17—C18—H18	119.9
C7—N1—Ni	122.61 (18)	C19—C18—H18	119.9
C8—N1—Ni	118.41 (16)	C14—C19—C18	120.5 (3)
O1—C1—C6	119.1 (2)	C14—C19—H19	119.8
O1—C1—C2	123.1 (2)	C18—C19—H19	119.8
C6—C1—C2	117.8 (2)	C21—C20—C25	119.2 (3)
C3—C2—C1	119.4 (2)	C21—C20—P1	117.7 (2)
C3—C2—C7	117.7 (2)	C25—C20—P1	123.0 (2)
C1—C2—C7	123.0 (2)	C20—C21—C22	120.4 (3)
C4—C3—C2	120.7 (3)	C20—C21—H21	119.8
C4—C3—H3	119.7	C22—C21—H21	119.8
C2—C3—H3	119.7	C23—C22—C21	120.1 (3)
C3—C4—C5	120.9 (3)	C23—C22—H22	120.0
C3—C4—Br1	119.5 (2)	C21—C22—H22	120.0
C5—C4—Br1	119.6 (2)	C22—C23—C24	120.0 (3)
C6—C5—C4	119.6 (3)	C22—C23—H23	120.0
C6—C5—H5	120.2	C24—C23—H23	120.0
C4—C5—H5	120.2	C23—C24—C25	120.5 (3)
C5—C6—C1	121.6 (3)	C23—C24—H24	119.7
C5—C6—H6	119.2	C25—C24—H24	119.7
C1—C6—H6	119.2	C20—C25—C24	119.7 (3)
N1—C7—C2	127.3 (2)	C20—C25—H25	120.2
N1—C7—H7	116.4	C24—C25—H25	120.2
C2—C7—H7	116.4	C31—C26—C27	119.0 (3)
C9—C8—C13	119.4 (2)	C31—C26—P1	120.8 (2)
C9—C8—N1	115.1 (2)	C27—C26—P1	120.2 (2)
C13—C8—N1	125.5 (2)	C28—C27—C26	120.6 (3)
C8—C9—C10	119.7 (2)	C28—C27—H27	119.7
C8—C9—S1	118.2 (2)	C26—C27—H27	119.7
C10—C9—S1	122.1 (2)	C29—C28—C27	119.7 (3)
C11—C10—C9	120.3 (3)	C29—C28—H28	120.2
C11—C10—H10	119.9	C27—C28—H28	120.2
C9—C10—H10	119.9	C30—C29—C28	120.0 (3)
C12—C11—C10	119.8 (3)	C30—C29—H29	120.0
C12—C11—H11	120.1	C28—C29—H29	120.0
C10—C11—H11	120.1	C29—C30—C31	120.9 (3)
C11—C12—C13	120.6 (3)	C29—C30—H30	119.6
C11—C12—H12	119.7	C31—C30—H30	119.6
C13—C12—H12	119.7	C30—C31—C26	119.7 (3)
C12—C13—C8	120.3 (3)	C30—C31—H31	120.1
C12—C13—H13	119.9	C26—C31—H31	120.1
			
O1—Ni—P1—C26	52.43 (11)	C8—C9—C10—C11	−2.2 (4)
N1—Ni—P1—C26	−120 (6)	S1—C9—C10—C11	176.5 (2)
S1—Ni—P1—C26	−134.10 (9)	C9—C10—C11—C12	0.5 (5)
O1—Ni—P1—C20	−65.88 (11)	C10—C11—C12—C13	1.1 (5)
N1—Ni—P1—C20	122 (6)	C11—C12—C13—C8	−0.9 (5)
S1—Ni—P1—C20	107.59 (9)	C9—C8—C13—C12	−0.8 (4)
O1—Ni—P1—C14	176.78 (12)	N1—C8—C13—C12	179.7 (3)
N1—Ni—P1—C14	5(6)	C26—P1—C14—C19	−117.6 (2)
S1—Ni—P1—C14	−9.76 (10)	C20—P1—C14—C19	−7.8 (3)
O1—Ni—S1—C9	−127.2 (4)	Ni—P1—C14—C19	111.0 (2)
N1—Ni—S1—C9	1.92 (11)	C26—P1—C14—C15	64.3 (2)
P1—Ni—S1—C9	−178.25 (9)	C20—P1—C14—C15	174.2 (2)
N1—Ni—O1—C1	11.1 (2)	Ni—P1—C14—C15	−67.1 (2)
S1—Ni—O1—C1	139.8 (4)	C19—C14—C15—C16	−1.1 (4)
P1—Ni—O1—C1	−168.8 (2)	P1—C14—C15—C16	177.0 (3)
O1—Ni—N1—C7	−5.1 (2)	C14—C15—C16—C17	−0.4 (5)
S1—Ni—N1—C7	−178.6 (2)	C15—C16—C17—C18	1.8 (5)
P1—Ni—N1—C7	167 (25)	C16—C17—C18—C19	−1.7 (5)
O1—Ni—N1—C8	172.73 (18)	C15—C14—C19—C18	1.2 (4)
S1—Ni—N1—C8	−0.74 (17)	P1—C14—C19—C18	−176.9 (2)
P1—Ni—N1—C8	−15 (6)	C17—C18—C19—C14	0.3 (5)
Ni—O1—C1—C6	170.00 (19)	C26—P1—C20—C21	−175.2 (2)
Ni—O1—C1—C2	−10.7 (4)	C14—P1—C20—C21	77.0 (2)
O1—C1—C2—C3	−178.6 (3)	Ni—P1—C20—C21	−49.2 (2)
C6—C1—C2—C3	0.7 (4)	C26—P1—C20—C25	3.8 (2)
O1—C1—C2—C7	1.7 (4)	C14—P1—C20—C25	−104.0 (2)
C6—C1—C2—C7	−179.0 (3)	Ni—P1—C20—C25	129.8 (2)
C1—C2—C3—C4	−0.7 (4)	C25—C20—C21—C22	2.2 (4)
C7—C2—C3—C4	179.0 (3)	P1—C20—C21—C22	−178.8 (2)
C2—C3—C4—C5	0.2 (5)	C20—C21—C22—C23	−0.9 (5)
C2—C3—C4—Br1	−178.3 (2)	C21—C22—C23—C24	−0.3 (5)
C3—C4—C5—C6	0.2 (5)	C22—C23—C24—C25	0.3 (5)
Br1—C4—C5—C6	178.7 (2)	C21—C20—C25—C24	−2.1 (4)
C4—C5—C6—C1	−0.1 (5)	P1—C20—C25—C24	178.9 (2)
O1—C1—C6—C5	179.0 (3)	C23—C24—C25—C20	0.9 (4)
C2—C1—C6—C5	−0.3 (4)	C20—P1—C26—C31	109.3 (2)
C8—N1—C7—C2	−179.0 (3)	C14—P1—C26—C31	−141.7 (2)
Ni—N1—C7—C2	−1.2 (4)	Ni—P1—C26—C31	−9.8 (3)
C3—C2—C7—N1	−175.1 (3)	C20—P1—C26—C27	−69.3 (2)
C1—C2—C7—N1	4.5 (5)	C14—P1—C26—C27	39.7 (2)
C7—N1—C8—C9	176.7 (2)	Ni—P1—C26—C27	171.56 (19)
Ni—N1—C8—C9	−1.2 (3)	C31—C26—C27—C28	−3.0 (4)
C7—N1—C8—C13	−3.7 (4)	P1—C26—C27—C28	175.6 (2)
Ni—N1—C8—C13	178.4 (2)	C26—C27—C28—C29	1.0 (5)
C13—C8—C9—C10	2.3 (4)	C27—C28—C29—C30	1.2 (5)
N1—C8—C9—C10	−178.1 (2)	C28—C29—C30—C31	−1.2 (6)
C13—C8—C9—S1	−176.5 (2)	C29—C30—C31—C26	−0.9 (5)
N1—C8—C9—S1	3.2 (3)	C27—C26—C31—C30	3.0 (5)
Ni—S1—C9—C8	−3.3 (2)	P1—C26—C31—C30	−175.6 (3)
Ni—S1—C9—C10	178.0 (2)		

### Hydrogen-bond geometry (Å, °)

**Table:**

*D*—H···*A*	*D*—H	H···*A*	*D*···*A*	*D*—H···*A*
C23—H23···S1^i^	0.93	2.84	3.620 (3)	142

Symmetry codes: (i) *x*+1, *y*, *z*.
